# Die periprothetische Fraktur beim Hüftgelenkersatz – Risikofaktoren und Therapie

**DOI:** 10.1007/s00132-025-04658-z

**Published:** 2025-05-21

**Authors:** Matthias Luger, Tobias Gotterbarm, Clemens Schopper

**Affiliations:** 1 Klinik für Orthopädie & Traumatologie, Kepler Universitätsklinikum GmbH, Johannes Kepler Universität Linz, Krankenhausstraße 9, 4020 Linz, Österreich; 2 Altenberger Straße 69, 4040 Linz, Österreich

**Keywords:** Acetabulum, Algorithmen, Femurfraktur, Hüftfraktur, Risikofaktoren, Acetabulum, Algorithms, Femoral fracture, Hip fracture, Risk factors

## Abstract

**Hintergrund:**

Periprothetische Acetabulumfrakturen stellen hauptsächlich ein Problem der zementfreien Implantationstechnik dar und treten in diesem Zusammenhang in etwa 3,6 % der primären und in bis zu 20,9 % der Revisionsfälle auf. Sobald eine Fraktursituation vorliegt, welche die Integrität der Beckenpfeiler betrifft, ist neben der Implantation einer Revisionspfanne zusätzlich eine diesbezügliche Stabilisierung mittels Plattenosteosynthese notwendig.

Periprothetische Femurfrakturen treten ebenfalls hauptsächlich bei Verwendung einer zementfreien Technik auf und stellen 0,4–6,8 % der Revisionen nach primärer Hüft-TEP-Implantation dar. Neben der zementfreien Technik stellen ein minimal-invasiver, ventral orientierter Zugang, weibliches Geschlecht, schlechte Knochenqualität, Alter > 75 Jahre sowie ein Revisionseingriff die häufigsten Risikofaktoren für die Entstehung einer PFF dar.

**Therapie:**

Je nach Grad der Stabilitätsgefährdung des Implantats können eine konservative oder eine operative Versorgung mittels Osteosynthese, Schaftwechsel oder einer Kombination beider Verfahren erfolgen.

## Abstract (en)

**Background:**

Periprosthetic acetabular fractures primarily pose a problem with cementless implantation techniques and occur in approximately 3.6% of primary and up to 20.9% of revision cases. When a fracture situation exists that affects the integrity of the pelvic columns, stabilization using plate osteosynthesis is necessary, in addition to the implantation of a revision cup.

Periprosthetic femoral fractures also primarily occur with cementless techniques and account for 0.4–6.8% of revisions after primary total hip arthroplasty. In addition to the cementless technique, the most common risk factors for the development of PFF are a minimally invasive, ventral approach, female gender, poor bone quality, age > 75 years, and revision surgery.

**Treatment:**

Depending on the degree of risk to the stability of the implant, conservative or surgical treatment can be carried out using osteosynthesis, stem replacement or a combination of both procedures.

Periprothetische Frakturen, egal ob im Bereich des Pfannenlagers oder des Femurs, stellen eine seltene aber dennoch unverändert schwerwiegende Komplikation in der modernen Endoprothetik dar, die zwingende Aufmerksamkeit vom orthopädisch-traumatologischen Behandlungsteam erfordert. Insbesondere aufgrund der steigenden Zahlen des Gelenkersatzes sowie der Erwartungshaltung an Implantate und Funktion muss diesem Problem in zunehmendem Ausmaß Aufmerksamkeit gewidmet werden. Grundsätzlich kann zwischen intra- und peri-/postoperativen Frakturen unterscheiden werden.

## Periprothetische Acetabulumfrakturen

Die Inzidenz intraoperativer periprothetischer Acetabulumfrakturen reicht von 0,3–5,4 %, wobei hier nochmals zwischen offensichtlichen und okkulten Fällen differenziert werden kann [1]. Während das Risiko bei Verwendung einer zementierten Technik als gering eingeschätzt werden kann (0,3 %), muss bei Verwendung einer zementfreien Technik deutlich häufiger mit dieser Komplikation gerechnet werden (5,4 %) [2, 3]. Grundsätzlich besteht bei Revisionseingriffen ein darüber hinaus erhöhtes Risiko (20,9 %) gegenüber primären Eingriffen (3,6 %) aufgrund des oftmals nicht vermeidbaren knöchernen Flurschadens, der zu einer zusätzlichen Schwächung des azetabulären Knochenstocks führt [3, 4]. Die Entstehung des intraoperativen Frakturtyps wird begünstigt durch gleichsam Unterfräsung wie auch Überfräsung, wobei das höchste Risiko der Entstehung im Moment der Implantatimpaktion auftritt, was die zementfreie Technik betrifft [1]. Diesbezüglich kann das Frakturrisiko reduziert werden, wenn „line to line“ gefräst wird [5]. Weitere Risikofaktoren – für die zementierte wie auch die zementfreie Technik gleichermaßen – stellen die dysplastische Koxarthrose, reduzierte Knochenqualität, Oversizing des Implantats sowie Implantatdurchmesser von 50 mm und kleiner dar [1, 6–8]. Was das Querschnittdesign zementfreier Pfannen betrifft, kann zwischen hemisphärischen, elliptischen und peripher verankernden Pfannen unterschieden werden. Hinsichtlich des intraoperativen Frakturrisikos in Bezug auf das verwendete Querschnittsdesign ist bekannt, dass elliptische Geometrien ein geringeres Risiko aufweisen als hemisphärische und peripher verankernde Geometrien [5, 9–12].

**Abb. 1:**
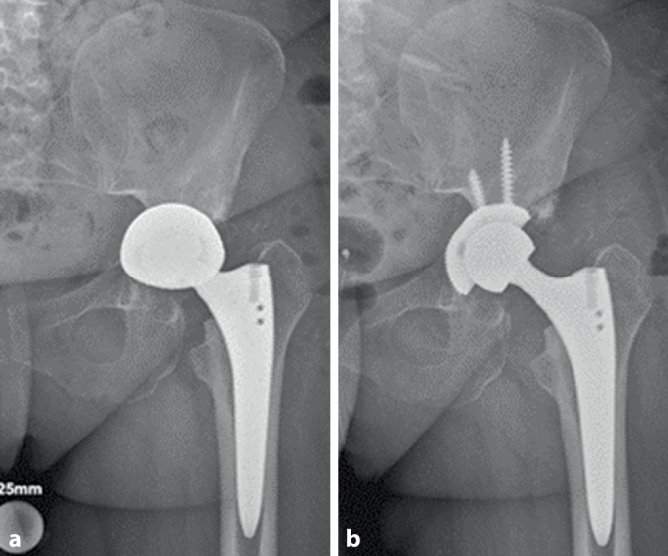
Überfräsung im Sinne eines medialen Wanddefekts und konsekutiver Pfannenlockerung (**a**). Wechsel auf verschraubte Pfanne bei intakter Zirkumferenz (**b**)

Wenn intraoperative Frakturen auftreten, dann am häufigsten entweder im Sinne einer medialen Wandläsion durch eine Überfräsung oder im Sinne einer superolateralen Zirkumferenzsprengung während der Implantatimpaktion ([9]; Abb. 1 und 2). Um eine hinreichende Einschätzung in Bezug auf die Implantatstabilität erreichen zu können, existieren diverse Klassifikationen, die eine diesbezügliche Einschätzung erlauben. Neben dem ursprünglichen Ansatz von Petersen und Lewallen, der lediglich zwischen 2 Varianten unterscheidet (stabil/instabil), lässt die häufig genutzte Weiterentwicklung unter Paprosky und Della Valle eine deutlich besserer Differenzierung zu (intraoperativ während Implantatimpaktion/intraoperativ während Implantatentfernung/traumatisch/spontan, Beckendiskontinuität) während die Variante von Pascarella et al. zum aktuellen Zeitpunkt die gelungenste Vereinbarung aus Vollständigkeit und Übersichtlichkeit bietet (intraoperativ: stabil/instabil; postoperativ: stabil/instabil durch Trauma bzw. instabil bereits davor) [13–15]. Auf Basis der bekannten Klassifikationen existieren Algorithmen, die den Anspruch erheben, einen entsprechenden Behandlungspfad für die jeweilige Fraktursituation auszuleuchten.

**Abb. 2:**
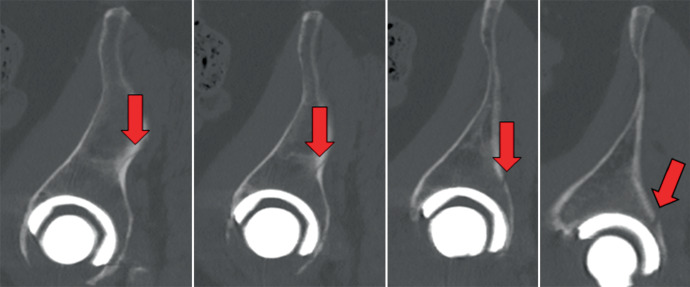
Superolaterale Iliumfraktur (*Pfeile*) nach Implantation einer Press-fit-Pfanne

## Periprothetische Acetabulumfrakturen: Behandlungsalgorithmus

**Abb. 3:**
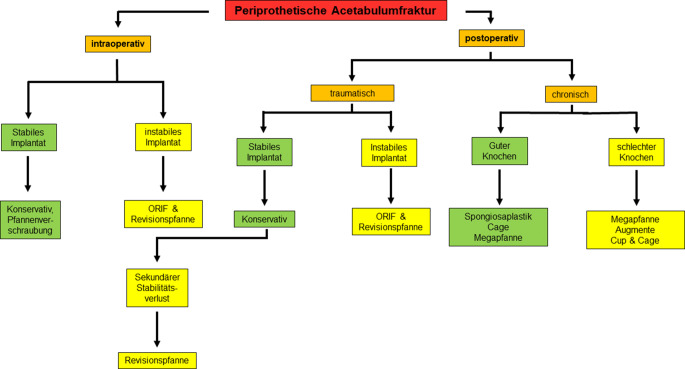
Algorithmus zur Behandlung von periprothetischen Acetabulumfrakturen; *ORIF* „open reduction and internal fixation“. (Modifiziert nach [2])

**Abb. 4:**
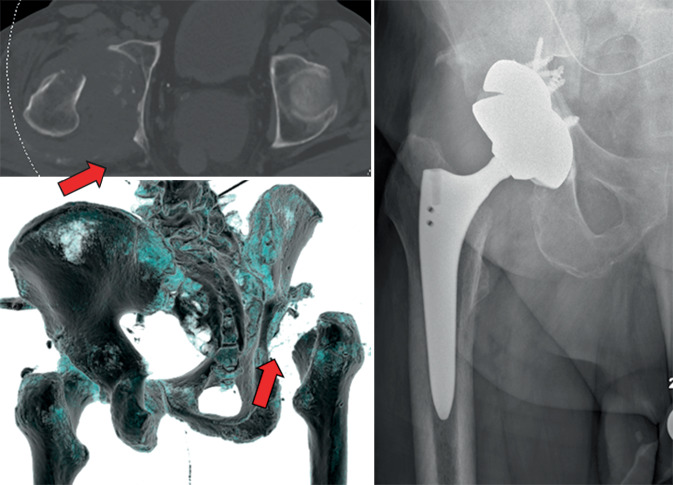
Acetabulumrekonstruktion mittels „Trabecular-metal“-Augmenten bei dorsalem Pfeilerdefekt (*Pfeile*)

**Abb. 5:**
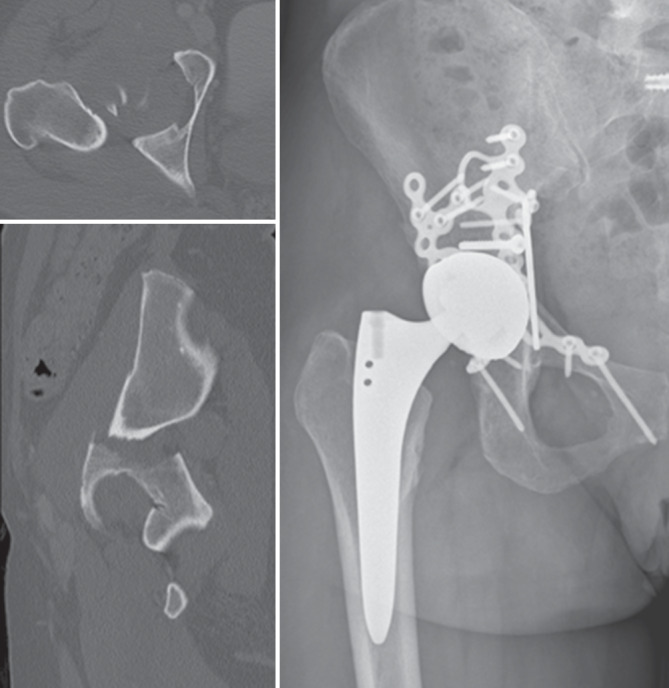
ORIF („open reduction and internal fixation“) und tripolare Press-fit-Pfanne bei kranialer Pfeilerfraktur und intakter Zirkumferenz

Unter den in der Literatur beschriebenen Algorithmen zeigt jener von Patsiogiannis et al. ein gutes Beispiel für ausführliche und gleichzeitig übersichtliche Pfadfindung zur Behandlung periprothetischer Acetabulumfrakturen ([2]; Abb. 3). Im Rahmen dieses Algorithmus wird grob unterschieden, ob die Fraktur intra- oder postoperativ entstanden ist und ob es sich um eine stabile oder eine instabile Situation handelt. Im Falle des postoperativen Frakturereignisses wird darüber hinaus zwischen traumatischen und chronischen Instabilitäten sowie guter und schlechter Knochenqualität unterschieden [2]. Als Faustregel kann gelten, dass im Falle von instabilen Fraktursituationen eine Plattenosteosynthese zusätzlich zur Implantation einer Revisionspfanne durchgeführt werden sollte. Hier muss vor allem unterschieden werden, ob einer der Beckenpfeiler und/oder die azetabuläre Zirkumferenz mitbetroffen sind. Ist die azetabuläre Zirkumferenz defizitär oder die Situation mit schlechter Knochenqualität vergesellschaftet, werden Augmente und „Cup-&-cage“-Systeme notwendig (Abb. 4). Ist einer der das Acetabulum bildenden Pfeiler von einer Fraktur betroffen, benötigt es eine Stabilisierung mittels – meistens – einer Plattenosteosynthese (Abb. 5; [4]).

## Periprothetische Femurfraktur

Auch periprothetische femorale Frakturen (PFF) stellen eine schwerwiegende Komplikation dar [16]. PFF können ebenfalls intra- oder postoperativ auftreten [2]. In der Literatur sind unterschiedliche Risikofaktoren für das Auftreten von PFF beschrieben, wobei zu den patientenspezifischen Risikofaktoren vor allem zunehmendes Alter, weibliches Geschlecht, Osteoporose oder rheumatoide Arthritis zählen ([16]; Tab. 1). Eine zementfreie Verankerung ist grundsätzlich mit einer höheren Rate an sowohl intra- als auch postoperativ auftretenden PFF assoziiert [16, 17]. Intraoperativ auftretende PFF sind bei zementfreier Technik als höher beschrieben (5,4 %) verglichen zu zementierter Technik (0,3 %) und signifikant höher bei Revisionseingriffen (20,9 %) verglichen mit primären Implantationen (3,6 %) [2, 3]. Postoperativ auftretende PFF sind mit einer Inzidenz von weniger als 1 % nach primärem Eingriff und mit bis zu 4 % nach Revisionseigriff beschrieben [2, 3, 18, 19]. Das Risiko für das Auftreten einer PFF ist mit 0,4–3,5 % in der Literatur beschrieben [2, 17, 20]. PPF sind der dritthäufigste Revisionsgrund und der zweithäufigste nach dem 4. Jahr nach Erstimplantation [2, 21–25] im Schwedischen Endoprothesenregister [2].

**Tab. 1:** Risikofaktoren für periprothetische Frakturen bei Implantation einer Hüfttotalendoprothese

Acetabulum	Femur
Zementfreie Verankerung	Zementfreie Verankerung
Chronischer Knochenverlust/Knochendefekte	Implantatdesign (Single Wedge/Double Wedge)
Osteoporose	Weibliches Geschlecht
Kleiner Durchmesser der Pfanne	Zunehmendes Alter
Oversizing	Osteoporose
Revision	Rheumatoide Arthritis
Dysplasie	Revision
Rheumatoide Arthritis	
Implantatdesign (hemisphärische Pfannengeometrie)	

Abdel et al. zeigten ein 14-mal häufigeres Auftreten von intraoperativen PFF bei zementfreien Implantaten sowie ein höheres Risiko für weibliche Patientinnen älter als 65 Jahre [17]. Postoperativ auftretende PFF sind bei zementfreien Implantaten als häufiger beschrieben, jedoch ohne Zusammenhang mit Alter und Geschlecht [17]. Hinsichtlich des Schaftdesigns zeigen Single-Wedge- und Double-Wedge-Designs bei zementfreien Schäften das höchste Risiko für das Auftreten von PFF [16]. Neuere Studien zeigen einerseits einen Vorteil durch den Einsatz von zementfreien Schäften mit Kragen [26, 27]. Ricotti et al. berichten über eine signifikant niedrigere Rate an PFF mit einem Schaft mit Kragen und Triple-Taper-Design mit 0,13 % verglichen mit 1,42 % für einen zementfreien Schaft ohne Kragen [26]. Im australischen Endoprothesenregister konnte durch den Einsatz von Kragenschäften über den direkt vorderen Zugang (DAA) eine signifikant niedrigere Gesamtrevisionsrate und eine signifikant niedrigere Rate an PFF innerhalb der ersten 6 Monate nach Implantation gezeigt werden [27].

Eine Möglichkeit zur Reduktion der Rate an PFF stellt grundsätzlich die zementierte Verankerung dar, wobei deren Anwendung von Zement großen geographischen Unterschieden unterliegt [28]. Es ist bekannt, dass die zementierte Versorgung bei weiblichen Patientinnen über 75 Jahren ein signifikant niedrigeres Risiko für das Auftreten von PFF und für das Risiko einer Revisionsoperation zeigt [29]. Während die Datenlage vor allem bei Patient:innen über 75 Jahren einen Vorteil der zementfreien Versorgung sieht, bestehen aber aufgrund der zunehmenden Anwendung des DAA technische Schwierigkeiten für die Zementiertechnik [30, 31]. Diesbezüglich werden vor allem kürzere Schaftdesigns empfohlen, da diese die Implantation erleichtern und ähnlich abschneiden hinsichtlich der bisher bekannten Kurzzeitergebnisse [30, 31]. Laboudie et al. zeigten eine Frakturrate von 0,48 % bei einem Follow-up von 2,6 Jahren mit einem neuen verkürzten Kerboull-Schaft über einen DAA bei Patient:innen über 70 Jahre mit „Line-to-line“-Zementiertechnik [30]. Weiters besteht auch bei zementierten Schäften ein Unterschied je nach verwendetem Implantat.

Zementierte Schäfte zeigen eine niedrigere Frakturrate im Vergleich zu zementfreien Schäften

Mabrouk et al. zeigten in einer rezenten Metaanalyse aus dem Jahr 2024, dass auch Unterschiede bezüglich der Frakturrate bei zementierten Schäften vorliegen [32]. Zementierte Schäfte nach dem Prinzip des „polished taper slip“ (PTS) zeigten ein höheres Risiko für das Auftreten von PFF im Vergleich zu zementierten Schäften nach dem Prinzip des „composite beam“ [32]. Somit zeigen zwar zementierte Schäfte eine niedrigere Frakturrate im Vergleich zu zementfreien Schäften, allerdings gibt es auch bei zementierten Schäften Unterschiede zwischen dem Auftreten von PFF. Auch der Zugangsweg wird als Risikofaktor für das Auftreten von PFF in der Literatur diskutiert [33–37]. Aggarwal et al. zeigten bei 3574 Hüft-TEP die niedrigste Frakturrate für den lateralen Zugang (2,7 %), während der PA und der DAA eine vergleichbare Rate zeigten (9,9 % vs. 8,4 %) [38]. In einer Fallserie von 684 Hüft-TEP implantiert über einen minimal-invasiven anterolateralen Zugang berichten Herndon et al. eine Frakturrate von 8,3 % innerhalb der ersten 90 Tage [34]. Nakai et al. berichten bei einer Fallserie von 103 Hüft-TEP über einen minimal-invasiven anterolateralen Zugang von einer signifikant höheren Rate an Frakturen des Trochanter major verglichen mit einer Kontrollgruppe bestehend aus 98 Hüft-TEP, implantiert über einen posterolateralen Zugang (2,9 % vs. 0,0 %; *p* < 0,01) [39]. Speziell bei minimal-invasiven Zugängen kann das Auftreten von PFF durch den Einsatz von Kurzschäften gesenkt werden [33, 40].

Bei zementfreier Technik über den DAA zeigte sich in einer Serie von 640 Fällen mit Extensionstisch eine Frakturrate von 6,8 % bei Implantation eines zementfreien Geradschaftes, während der Einsatz von zementfreien Kurzschäften das Auftreten von PFF mit 1,6 % signifikant reduzierte (*p* = 0,027) [33]. Der Einsatz von Kurzschäften zeigte auch beim minimal-invasiven anterolateralen Zugang eine signifikant niedrigere Rate an PFF innerhalb des ersten Jahres nach Implantation im Vergleich zur Implantation eines zementfreien Geradschaftes über einen transglutealen Zugang (1,7 % vs. 3,2 %, *p* = 0,015) [40]. Speziell das Auftreten von Vancouver-A-Frakturen im Sinne von Trochanter- oder Trochanterspitzenfrakturen kann durch den Einsatz von Kurzschäften bei minimal-invasiven Zugängen gesenkt werden [33, 40].

Die Vancouver-Klassifikation ist weiterhin die gebräuchlichste Klassifikation

**Abb. 6:**
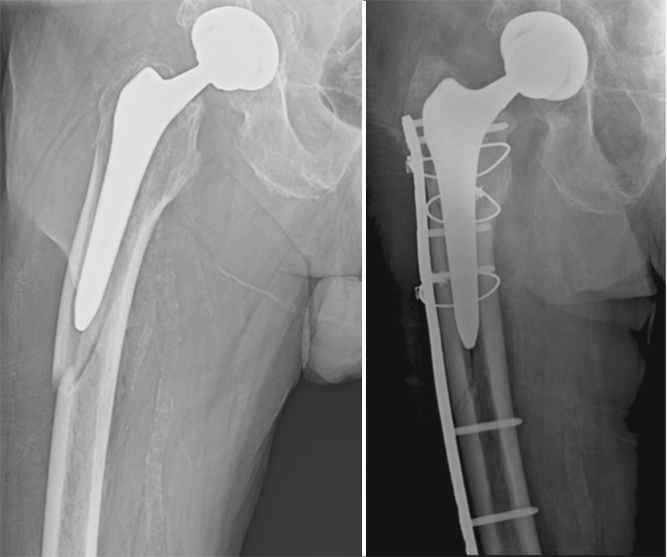
Vancouver-B1-Fraktur mit Implantaterhalt und ORIF („open reduction and internal fixation“) mittels Plattenosteosynthese und Cerclagen

Die Klassifikation der intra- und postoperativen PFF wird häufig mit der Vancouver-Klassifikation, beschrieben von Duncan und Masri, durchgeführt und stellt weiterhin eine der am häufigsten verwendeten Klassifikationen zur Einteilung von PFF dar [2, 41; Abb. 6, 7 und 8]. Diese wurde 2004 angepasst und 2009 mit Strategien zur Behandlung beziehungsweise Revision ergänzt [42, 43]. Die Vancouver-Klassifikation kann für intraoperative und postoperative PFF angewendet werden [43–45]. Eine neuere erweiterte Klassifikation für PFF stellt das „Unified Classification System“ dar, das für alle großen Gelenke verwendet werden kann [46]. Die Vancouver-Klassifikation mit der Unterteilung in A‑, B- und C‑Frakturen ist weiterhin die gebräuchlichste Klassifikation und ist die Grundlage für die Klassifikation von PFF und deren Behandlung [2, 47].

**Abb. 7:**
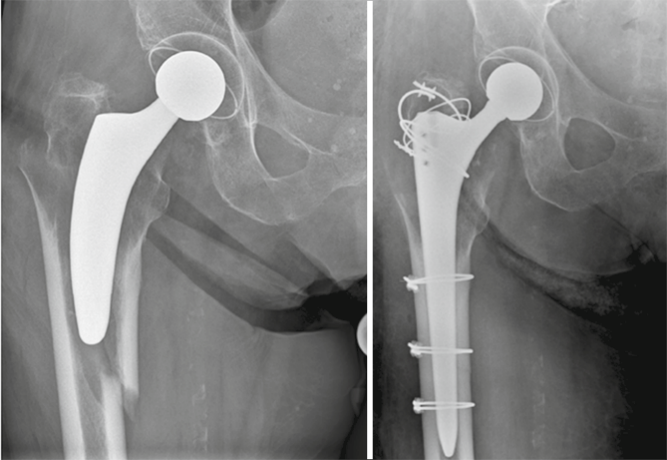
Vancouver-B2/AG-Fraktur mit zementfreiem Schaftwechsel und Cerclagenosteosynthese

**Abb. 8:**
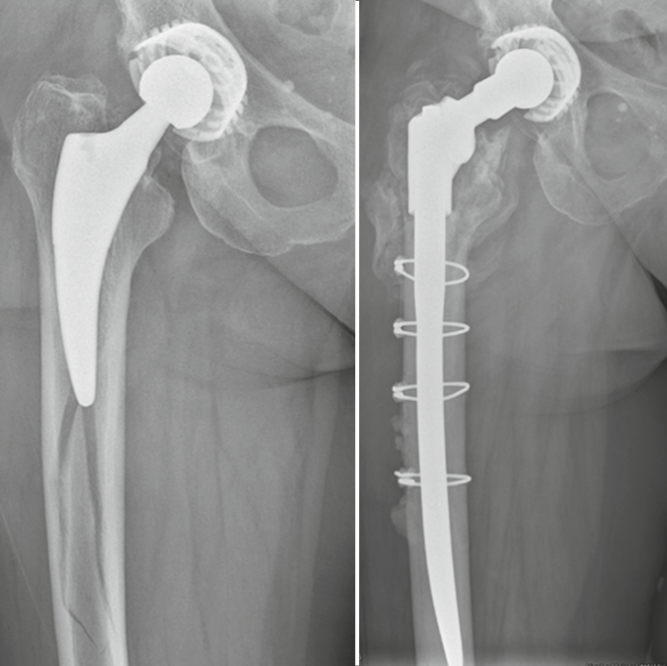
Vancouver-B2/AG/A_L_-Fraktur mit zementiertem, modularem Schaftwechsel

## Periprothetische Femurfraktur: Behandlungsalgorithmus

**Abb. 9:**
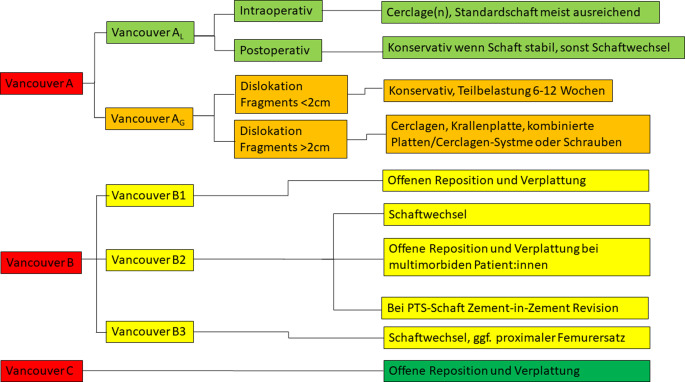
Algorithmus zur Behandlung von periprothetischen Femurfrakturen; *PTS* „polished taper slip“

Unverschobene postoperative PFF können konservativ behandelt werden, ebenso wenn es sich um multimorbide Patient:innen handelt ([48]; Abb. 9). Bei einer Dislokation des Fragments über 2 cm sollte aufgrund zu erwartender Folgebeschwerden wie Schmerzen oder einer Schwächung der pelvitrochantären Schlinge eine operative Behandlung erfolgen [48]. Hierfür stehen Cerclagen, Krallenplatte, kombinierte Platten-Cerclagen-Systeme oder Schrauben zur Verfügung [48]. A_L_-Frakturen sind seltener und können je nach Fragmentgröße und Dislokation zu einem Verlust des medialen Supports des Schaftes führen [48]. Postoperativ können viele A_L_-Frakturen konservativ behandelt werden, während intraoperativ auftretende A_L_-Frakturen meistens mittels Cerclagen stabilisiert werden und der Originalschaft belassen werden kann [2]. Dieses Frakturmuster wurde auch als Clamshell-Fraktur beschrieben und kann bei Verwendung oder Wechsel auf einen diaphysär verankernden Schaft bei medialem Defekt < 40 % der Schaftlänge auch ohne Cerclage belassen werden [49].

Vancouver-B-Frakturen sind PFF rund um den Prothesenschaft und werden in aller Regel chirurgisch behandelt, wobei zwischen fester (Vancouver B1) und lockerer Schaftverankerung (Vancouver B2) unterschieden wird [2, 48]. Im Falle einer Vancouver-B1-Fraktur erfolgt bei festem Prothesenschaft eine offene Reposition und Osteosynthese [2, 48]. Als Osteosynthesematerial stellt die Verplattung in Kombination mit Cerclagen die häufigste chirurgische Behandlungsoption dar, wobei auch Strutgrafts unterstützend eingesetzt werden [2]. Vancouver-B2-Frakturen sind mit lockerer Schaftkomponente charakterisiert und benötigen grundsätzlich einen Revisionseingriff mit Schaftwechsel [2, 48]. Im Anschluss an eine Reposition mit zum Beispiel Cerclagen erfolgt die Implantation einer ausreichend langen Monoblock- oder modularen Schaftkomponente um die Fraktur distal ausreichen zu überbrücken [2]. Additiv können auch bei Vancouver-B2-Frakturen Platten oder Struts eingesetzt werden [2].

Zementierte Schäfte nach dem PTS-Prinzip können trotz B2-Fraktur mit offener Reposition und Osteosynthese behandelt werden, wobei gegebenenfalls eine Zement-in-Zement-Revision durchgeführt werden kann [2]. Hierbei wird nach offener Reposition und Osteosynthese in den bestehenden Zementköcher eine kleinere Schaftgröße einzementiert. Bei multimorbiden und von der Mobilität sehr eingeschränkten Patient:innen kann auch bei Vancouver-B2-Fraktur zur Reposition der Fraktur trotz lockerer Schaftkomponente eine Osteosynthese erfolgen [50, 51]. Damit kann die Fraktur stabilisiert werden bei geringerer Operationszeit und niedrigerem Operationsrisiko [2].

Bei der Vancouver-B3-PFF besteht eine lockere Schaftkomponente im Sinne einer B2-Fraktur jedoch mit deutlich schlechterer Knochenqualität [2]. Hierbei ist grundsätzlich die Revision der Schaftkomponente mittels Schaftwechsel indiziert, wobei hier proximale femorale Allografts gegebenenfalls notwendig sind bzw. sogar ein proximaler Femurersatz notwendig sein kann [2]. Vancouver-C-PFF sind als Frakturen unterhalb der Prothesenspitze klassifiziert und werden mit offener Reposition und Plattenosteosynthese behandelt, da hierbei von einer festen Schaftkomponente ausgegangen werden kann [2, 48]. Grundsätzlich ist bei diesen Frakturen auch eine ausreichende Überbrückung des distalen Prothesenanteils um die gesamte Diaphyse zu überbrücken bis über den distalen Anteil der femoralen Komponente, wobei proximal die Platte mit Cerclagen oder Schrauben verankert wird [2].

## Fazit für die Praxis

- Periprothetische Acetabulumfrakturen sind hauptsächlich ein Problem der zementfreien Implantationtstechnik.
- Sobald eine Fraktursituation vorliegt, welche die Integrität der Beckenpfeiler betrifft, ist neben der Implantation einer Revisionspfanne zusätzlich eine Stabilisierung mittels Plattenosteosynthese notwendig.
- Periprothetische Femurfrakturen treten ebenfalls hauptsächlich bei Verwendung einer zementfreien Technik auf und stellen mit 0,4–6,8 % den dritthäufigsten Revisionsgrund nach primärer Hüfttotalendoprothese dar.
- In Anlehnung an die Vancouver-Klassifikation, welche die nach wie vor am häufigsten verwendetet Klassifikation darstellt, werden Frakturen nah (A), auf Höhe (B) und fern des Prothesenschaftes (C) unterschieden.
- Während der Typ A größtenteils konservativ behandelt werden kann, muss bei Typ B unterschieden werden, ob der Prothesenschaft fest verankert ist oder nicht. Typ C Frakturen werden größtenteils verplattet.

## Abkürzungen

DAA: Direkter vorderer Zugang
ORIF: „Open reduction and internal fixation“
PA: Hinterer Zugang
PFF: Periprothetische Femurfraktur
PTS: „Polished taper slip“
TEP: Totalendoprothese
